# High-Power Battery Electrodes Fabricated by Acupuncture-Inspired Microneedle Processing

**DOI:** 10.1021/acsami.4c11834

**Published:** 2024-10-03

**Authors:** Chun-Yang Kang, Le-Yen Lin, Thao Nguyen, Chia-Chin Chen, Jeng-Kuei Chang, Tzu-En Lin, Yu-Sheng Su

**Affiliations:** † Industry Academia Innovation School, National Yang Ming Chiao Tung University, Hsinchu 300093, Taiwan; ‡ Department of Chemical Engineering, National Taiwan University, Taipei 106319, Taiwan; § International College of Semiconductor Technology, National Yang Ming Chiao Tung University, Hsinchu 300093, Taiwan; ∥ Department of Materials Science and Engineering, National Yang Ming Chiao Tung University, Hsinchu 300093, Taiwan; ⊥ Institute of Applied Mechanics, National Taiwan University, Taipei 106319, Taiwan

**Keywords:** low-tortuosity, porous electrodes, diffusion coefficients, ion transport kinetics, concentration polarization, rate performance

## Abstract

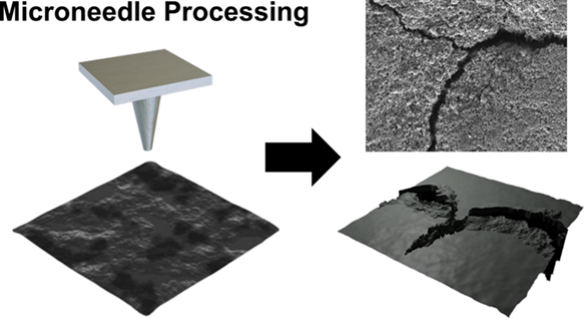

Advancing battery electrode performance is essential for high-power applications. Traditional fabrication methods for porous electrodes, while effective, often face challenges of complexity, cost, and environmental impact. Inspired by acupuncture, here we introduce an eco-friendly and cost-effective microneedle process for fabricating lithium iron phosphate electrodes. This technique employs commercial cosmetic microneedle molds to create low-curvature holes on electrode surfaces, significantly enhancing electrolyte infiltration and ion transport kinetics. The punctured electrodes were prepared and characterized, with comparisons to pristine electrodes conducted using scanning electron microscopy, 3D metallurgical microscopy, and detailed electrochemical evaluations. Our results show that the microneedle-processed electrodes exhibit superior rate performance and diffusion properties. Simulations and experimental data reveal that the low-curvature holes reduce Li-ion concentration polarization and improve Li-ion transport within the electrode. This enhancement leads to higher specific capacities and better rate capabilities in the punctured electrodes. The findings highlight the potential of this innovative microneedle technique for large-scale production of high-performance electrodes, offering a promising avenue for the development of high-power-density batteries.

## 1 Introduction

The development of high-performance battery electrodes is crucial for advancing energy storage technologies, particularly for applications demanding high power density. Porous electrodes are essential for high-power applications because they provide enhanced pathways for ion transport and improve electrolyte infiltration.^1−4^ These characteristics are vital for reducing ion transport resistance, minimizing concentration polarization, and ensuring uniform current distribution throughout the electrode. The increased surface area of porous electrodes facilitates faster charge and discharge rates, thereby improving the overall performance of the battery in high-power applications. Traditional fabrication methods for porous battery electrodes, such as freeze-drying,^5^ magnetic field assistance,^6^ 3D printing,^7^ laser drilling,^8^ and the template method,^9−11^ have shown various degrees of success in enhancing ion transport and electrochemical performance. However, these techniques often involve complex processes, high costs, or environmental concerns.

Inspired by the ancient practice of acupuncture in traditional oriental medicine, we introduce a novel, green, and low-cost microneedle process for fabricating battery electrodes. This approach not only mirrors the therapeutic principles of acupuncture by creating microholes to facilitate better internal flow but also seamlessly integrates into existing battery manufacturing processes. By employing commercial cosmetic microneedle molds, our method creates low-curvature holes on electrode surfaces, significantly improving electrolyte infiltration and ion transport kinetics. In this study, we demonstrate the efficacy of the acupuncture-inspired microneedle process in producing lithium iron phosphate (LFP) electrodes with enhanced performance characteristics. We detail the preparation and characterization of these electrodes, including the fabrication of punctured and pristine versions, their structural analysis using scanning electron microscopy (SEM) and 3D metallurgical microscopy, and comprehensive electrochemical evaluations. Our findings indicate that the microneedle-processed electrodes exhibit superior rate performance and diffusion properties compared to their pristine counterparts, highlighting the potential of this innovative technique for large-scale, high-power battery applications.

## 2 Experimental Section

### 2.1 Microneedle-Processed Electrode Fabrication

*N*-Methyl-2-pyrrolidone (NMP) was used as the solvent to prepare the electrode slurry. The slurry consisted of LFP (Green Energy Electrode) as the active material, carbon black (Super P) as the conductive additive, and polyvinylidene fluoride (PVDF 761-A) as the binder. Additionally, multiwalled carbon nanotubes (MWCNTs) were dispersed in the NMP solvent. The weight ratio of the materials was LFP: Super P: PVDF = 90:4:6, with an additional 1 wt % MWCNT. The well-mixed, glossy LFP slurry was applied onto flat aluminum foil using a doctor blade. The coated electrodes were then placed in a circulating oven at 30 °C for 1 h, resulting in a semidry state with reduced surface glossiness. In this condition, the electrodes retained a degree of plasticity, which allowed them to maintain a porous structure after the subsequent perforation. Afterward, the electrodes were removed from the oven and subjected to a microneedle process using a commercial cosmetic microneedle mold, creating indentations on the electrode surface. To detach the microneedle head from the cartridge, NMP was used to remove the adhesive. Following the microneedle process, the electrodes were returned to the oven for an additional 1.5 h of drying. Finally, the electrodes were placed in a vacuum oven at 120 °C for 12 h overnight to completely remove any remaining solvent. The microneedle process resulted in visible microholes on the electrode surface, which were further processed by calendering to ensure uniformity, creating the punctured electrodes. The areal and packing densities were controlled at 6.6 or 15.7 mg cm^–2^ and 1.3–1.4 g cm^3^, respectively. The preparation process for the pristine electrodes was identical to that of the punctured electrode, except for the omission of the microneedle processing step.

### 2.2 Microstructure Characterizations

Both the LFP electrode and microneedle mold were first platinum-coated to enhance the conductivity. Subsequently, the samples were examined using an SEM (Hitachi SU-8010) with an operating voltage set at 15 kV to observe the surface morphology and structure. A metallurgical microscope (Motic PA53MET) equipped with a *Z*-axis motorized module and 3D software was used to provide 3D topography of the electrodes. This setup allowed for a detailed observation of the irregular low-curvature hole surface morphology and depth. Different colors indicated various heights, with the highest surface point set to 0 μm, enabling accurate assessment of the hole depths in the electrodes.

### 2.3 Cell Assembly and Rate Capability Assessments

The pouch cell assembly was carried out in a dry room with a dew point below–35 °C. The LFP electrode was connected using an aluminum tab, while the lithium metal negative electrode was connected using a nickel tab, with a separator (Celgard 2325) placed between them. The layers were stacked and enclosed in aluminum laminate film packaging. The assembled pouch cell was then placed in a circulating oven at 40 °C for 1 h to remove moisture. The tabbed ends were welded to seal the pouch. Afterward, the cell was transferred to a glovebox with ultralow O_2_ and H_2_O levels, where the electrolyte (1 M lithium hexafluorophosphate in ethylene carbonate/ethyl methyl carbonate (1:2 v/v; Novolyte)) was injected. Once the excess air was removed, the final side was welded, completing the pouch cell assembly. The assembled pouch cells were subjected to rate and cycling tests using a battery tester (Lanhe CT3002A). Prior to testing, the cells were rested for 10 h and then charged/discharged at a rate of 0.1C for the first three cycles to activate the battery, with the 1C capacity for LFP set at 155 mAh g^–1^. During the cycling tests, the cells were first charged to 4.2 V, followed by a 10 min rest period, and then discharged to 2 V. After completing three cycles at 0.1C, the charge/discharge rate was increased to 1C. The rate performance test involved cycling the cells five times at each different C rate.

### 2.4 Electrochemical Characterizations

Cyclic voltammetry (CV) measurements were performed using a potentiostat (BioLogic SP-50e) at various scan rates, scanning up to 4.2 V and then back to 2.0 V. Electrochemical impedance spectroscopy (EIS) measurements were also conducted with the potentiostat over a frequency range of 1 MHz to 10 mHz, applying an AC voltage of 10 mV. For DC polarization, a constant current of 0.05 mA was applied for 2 h, followed by a rest period until the slope of the voltage versus time curve was less than 0.1 mV h^–1^. This process was repeated until the voltage reached or exceeded 4.2 V. The discharge process mirrored this approach until the slope was greater than 0.1 mV h^–1^, continuing until the voltage dropped to 2.0 V. The galvanostatic intermittent titration technique (GITT) test involved applying a positive current pulse at 0.1C, followed by a 10 min relaxation period. This cycle was repeated until the battery voltage reached 4.2 V for full charge. During discharge, the same process with a negative current pulse was followed until the battery was fully discharged to 2.0 V.

## 3 Results and Discussion

**Figure 1:**
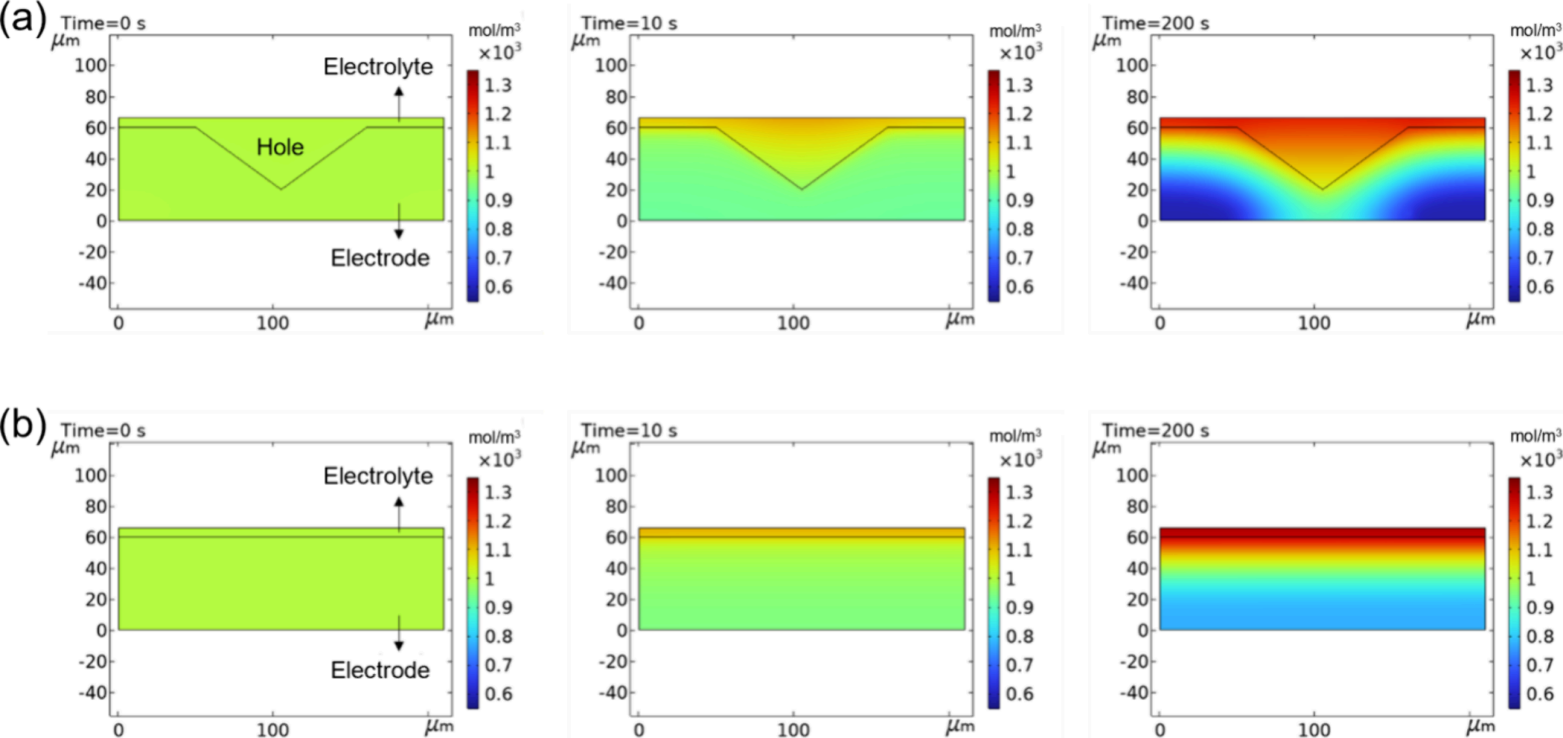
Simulation results demonstrating Li-ion transport dynamics in the (a) punctured electrode and (b) pristine electrode without any holes. Compared to the flat electrode, the punctured electrode reduces the degree of Li-ion concentration polarization due to the additional electrolyte infiltration pathways provided by the low-curvature holes.

To investigate the factors enhancing the battery performance of LFP electrodes with low-curvature holes, simulations were conducted using COMSOL Multiphysics software. The relevant parameters are detailed in Table S1, and Figure 1 displays the simulation results. The Li-ion concentration was compared under different discharge times for the punctured electrode and flat pristine electrode with low-curvature holes. In the case of the pristine electrode, a uniform 1-D concentration gradient is observed during discharge. Significant concentration differences at various electrode depths start to emerge after discharging for more than 200 s, indicating restricted ion transport kinetics.^12^ In contrast, the presence of low-curvature holes mitigates the degree of Li-ion concentration polarization at the upper regions of the LFP electrode, which is especially notable during prolonged discharge times. This phenomenon arises because low-curvature holes provide additional electrolyte permeation channels, enhancing ion transport kinetics.^1,13^ Notably, due to the presence of a cavity, the region near the tip of the low-curvature hole has a higher Li-ion concentration than other locations at the same depth. This observation highlights the significant impact of low-curvature holes on Li-ion distribution and ion transport within the electrode.

**Figure 2:**
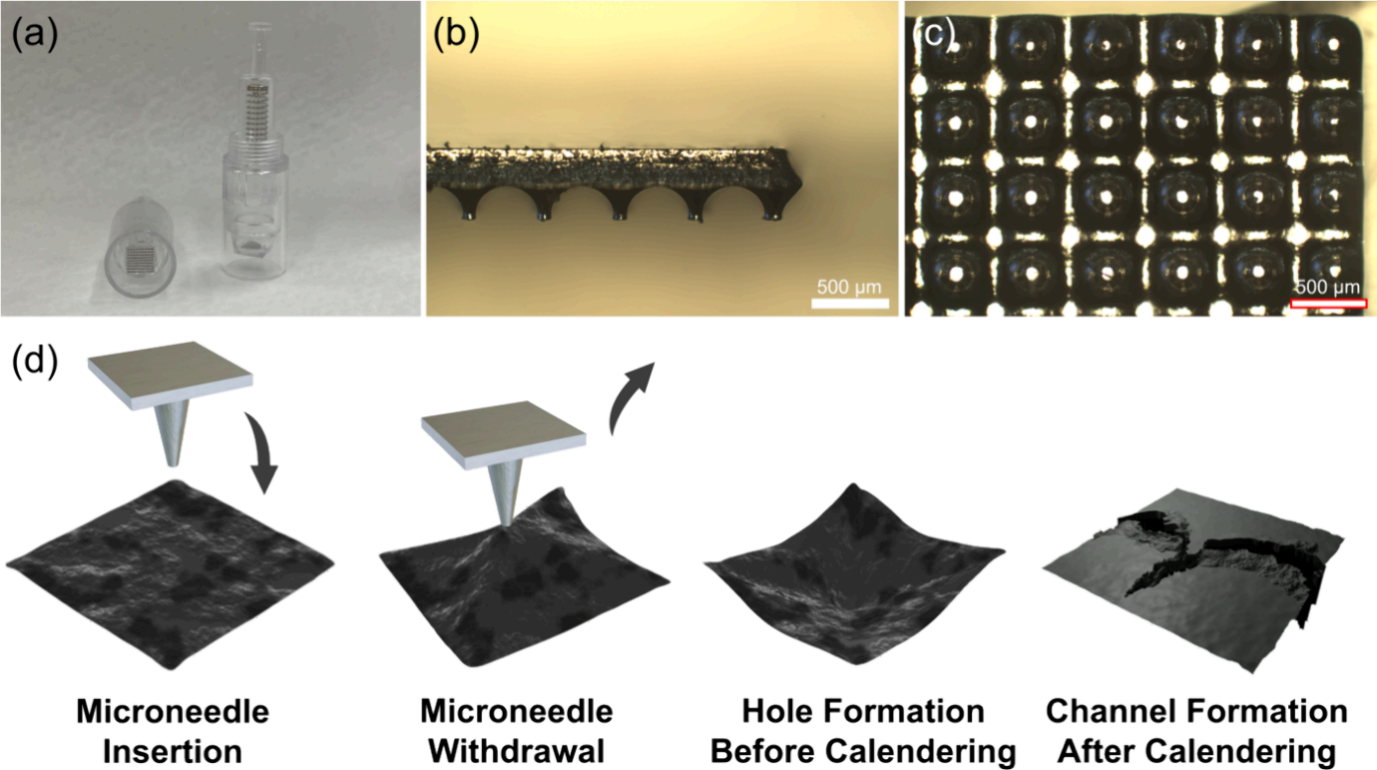
(a) Microneedle cartridges. (b) Cross-sectional and (c) top-view images of microneedle arrays. (d) Punctured electrode fabrication steps via microneedle processing. Low-curvature holes in the electrode further develop into channel-like pathways after calendering.

Commercially available, low-cost microneedle cartridges made of chrome steel, as shown in Figure 2a–c, were used to create channels during electrode fabrication. After completing the electrode coating, the stamping process using a microneedle was initiated, as shown in Figure 2d. When the electrode was in a semidry state with a lower surface glossiness, it was removed from the convection oven. A pressure of 3.36 kPa was applied to the microneedle mold to emboss and perforate the electrode surface, resulting in indentations. Due to the semidry state of the electrode, it exhibited some stickiness during the microneedle withdrawal step. After the microneedle process, the electrode was returned to the vacuum oven for drying to completely remove the solvent. Once fully evaporated, the electrode surface displayed microscale holes visible to the naked eye. These holes were then further deformed by roller calendering, forming low-curvature channels. In order to compare the electrochemical performance, the areal loading and packing density were controlled to be the same for both the punctured and pristine electrodes in the subsequent analysis.

**Figure 3:**
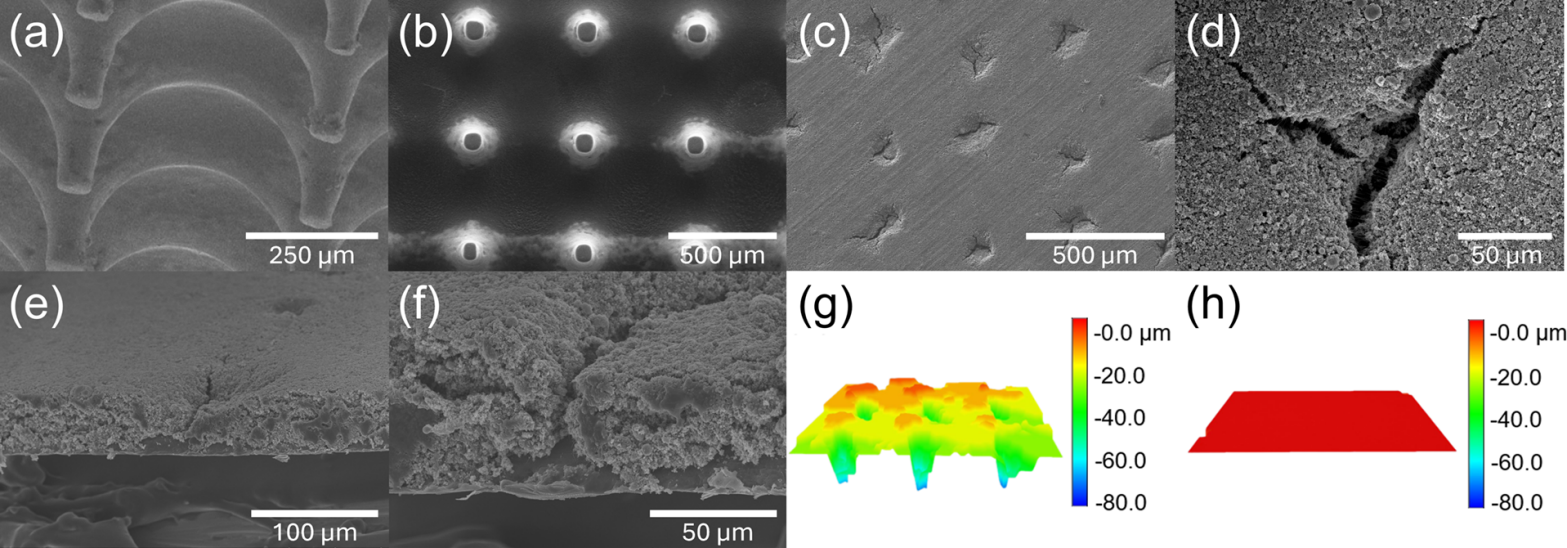
SEM images of microneedle arrays from (a) side view and (b) top view. SEM images of the punctured LFP electrode from (c,d) top view and (e,f) side view. 3D reconstruction images of the (g) punctured LFP electrode and (h) pristine LFP electrode.

SEM imaging was employed to analyze the dimensions and morphology of the microneedle arrays and the structure of the punctured electrode. Figure 3a,b shows the microneedle structure, which has a depth of approximately 150 μm and a spacing of about 500 μm, featuring a flat-topped funnel shape. Figure 3c,d presents SEM microstructural top views of the punctured electrode, where the holes exhibit a radial pattern emanating from the center. This pattern results from the microneedle piercing the semidry electrode surface and being withdrawn, causing adhesion effects. The holes, along with their shapes, sizes, and crack widths, are not identical. The cross-sectional SEM images (Figure 3e,f) reveal that the holes formed by the microneedle process are not vertically isotropic but instead exhibit a larger surface opening tapering into a vertical funnel shape. This characteristic is attributed to the microneedle structure used in the experiment. A 3D stereoscopic image reconstructed from a 3D metallurgical microscope scan (Figure 3g) shows the red areas representing the electrode surface and the blue areas indicating the bottom. Clearly, after the microneedle process, the depth of each hole can reach up to 80 μm in the punctured electrode. These funnel-shaped channels enhance electrolyte permeation and Li-ion diffusion.^14^ In contrast, without the microneedle process, the pristine electrode surface remains smooth and flat (Figure 3h).

**Figure 4:**
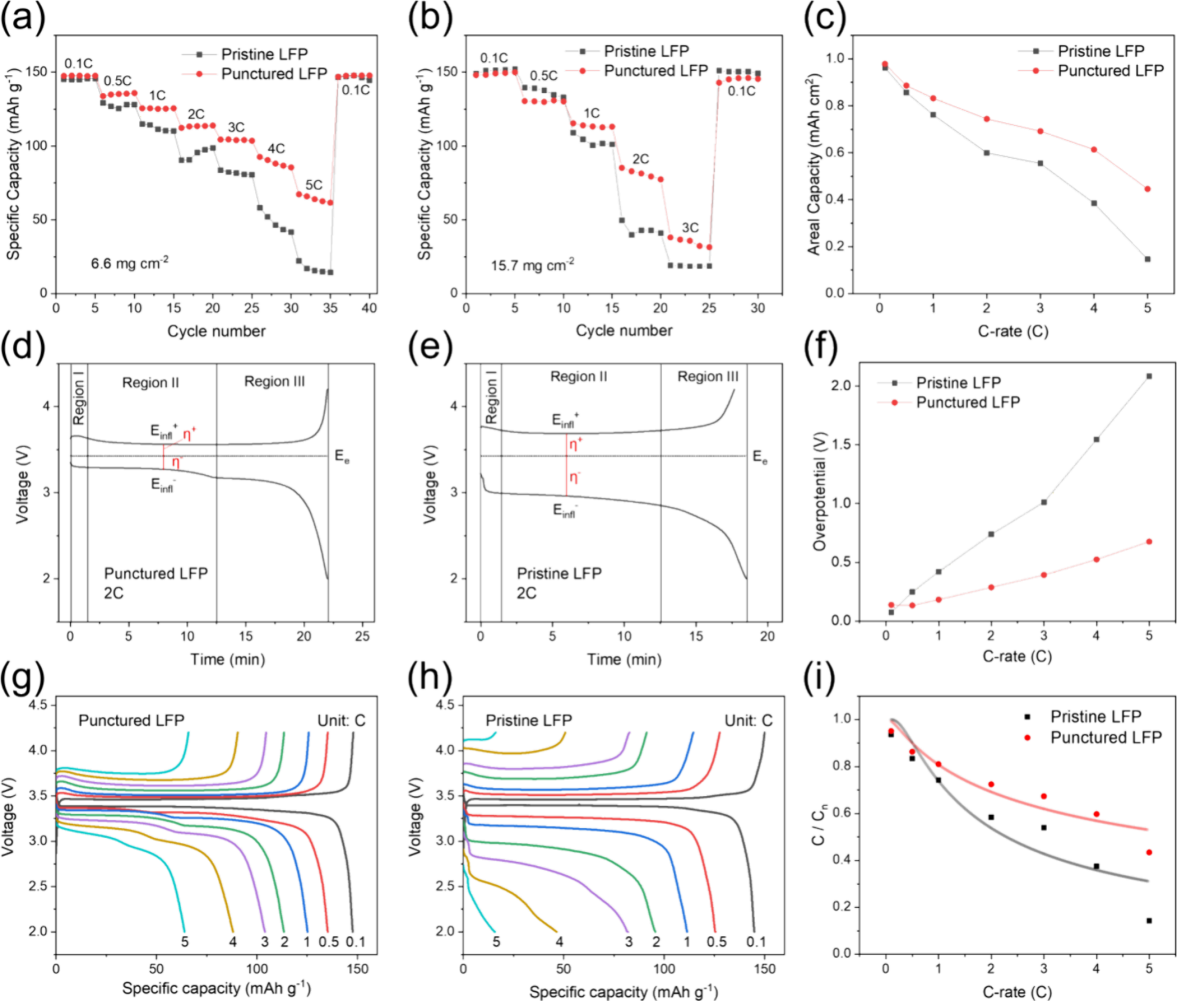
Rate performance of the punctured and pristine LFP electrodes with an areal density of (a) 6.6 mg cm^–2^ and (b) 15.7 mg cm^–2^. (c) Areal capacity versus C-rate profiles. Overpotential analysis of the (d) punctured LFP electrode and (e) pristine LFP electrode at 2C. (f) Overpotential versus *C*-rate profiles. Charge and discharge profiles of the (g) punctured LFP electrode and (h) pristine LFP electrode at various *C*-rates. (i) *C*/*C*_n_ value versus *C*-rate profiles.

As electrode thickness and areal density increase, rate performance generally declines. Rate capability tests were conducted to investigate whether low-curvature holes can enhance electrochemical performance under various current rates. Figure 4a,b depicts the specific capacities of LFP electrodes with two different areal densities. At the 0.1C rate, the punctured electrode with an areal density of 6.6 mg cm^–2^ exhibits an average capacity of 145 mAh g^–1^, while at the 3C rate, the discharge capacity is still high at 104 mAh g^–1^. For the thicker electrode with an areal density of 15.7 mg cm^–2^, the specific capacities of the punctured electrode at 1C and 2C rates are 114 and 81 mAh g^–1^, respectively. Compared to the pristine electrode, the punctured electrode demonstrates more promising rate performance across different rates, with a retention ratio exceeding 50% at 2C, which is significantly higher than that of the pristine electrode tested. When the current rate is reduced to 0.1C, both electrodes with different areal densities show good capacity recovery behavior. The punctured electrode (6.6 mg cm^–2^) outperforms the pristine electrode in areal capacity retention at various current rates, leading to over 90% at 0.5C, over 85% at 1C, over 76% at 2C, over 71% at 3C, over 61% at 4C, and over 45% at 5C (Figure 4c). To determine whether the microneedle process affects the cycle life of the electrode, the capacity retention of the punctured and pristine LFP electrodes at 1C is compared in Figure S1. The results indicate that the punctured electrode exhibits excellent capacity retention and Coulombic efficiency, comparable to the pristine electrode. This suggests that the microneedle process effectively enhances power density without compromising cycle stability. Notably, the low-curvature holes remain intact after cycling, as exhibited in Figure S2, which facilitates smooth ion diffusion. Table S2 provides a comparison of recent techniques for fabricating thick and porous LFP electrodes. The microneedle processing method stands out as a scalable approach that can be seamlessly integrated into standard battery electrode manufacturing without requiring modifications to the electrode composition or the introduction of specialized tools and external fields (e.g., acoustic or magnetic). Furthermore, the microneedle method supports a high active material content (90 wt %) while still delivering good rate capability and stable cycle life.

Figure 4d,e investigates the characteristics of voltage profile and overpotential by charging and discharging the punctured and pristine LFP electrodes at 2C, with the voltage–time curves plotted accordingly. The curves are divided into three regions, showing a steep initial rise or decline that gradually levels off (Region I), a plateau region (Region II), and the slope of the curve gradually increases or decreases (Region III). An inflection point can be found in Region II, which can be identified in the first derivative of a galvanostatic curve as a function of potential.^15^ The total overpotential of the cell, η, is calculated as the difference between the measured voltage of the cell at the inflection point, *E*_infl_, and the global equilibrium potential, *E*_e_, at 3.43 V.^15,16^ The formula can be written as
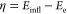
1

A lower total overpotential implies better energy efficiency and reduced polarization (Table S3), which explains why the punctured LFP electrode exhibits superior high-power charge–discharge performance compared to the pristine electrode. By calculating the overpotential from the charge/discharge plateau difference and plotting it against various rates in Figure 4f, the punctured electrode exhibits significantly lower overpotential compared to the pristine electrode. This reduction in overpotential signifies less polarization and improved electron/ion transport performance, thereby enhancing the rate capability.^17^Figure 4g,h shows that as the charge/discharge rate increases from 0.1C to 5C, the overpotential gap of the punctured electrode widens more slowly than that of the pristine electrode. At 5C, the voltage plateau difference between the two electrodes becomes highly pronounced.

Next, a semiempirical model was used to fit the capacity at different rates, allowing us to derive an advanced comparison of rate capability. This can be determined by the relaxation of the rate-limiting process as follows^18^:
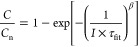
2Here, *C* is the first-cycle discharge capacity at different rates, *C*_n_ is the nominal capacity of LFP, and τ_fit_ is the time constant of the rate-limiting process, which is related to the inverse of the current rate (*I*), expressed as *C*-rate. The parameter β is an empirical value from the semiempirical model, where β greater than 1 indicates an accelerated nonexponential relaxation process, and β less than 1 indicates a delayed nonexponential relaxation process.^18^ By fitting the relationship between  and the *I* using the above formula (Table S4), it is found that when , the discharge time (the inverse of *I*) is much longer than the time constant of the rate-limiting process. This implies that lithium ions have enough time to intercalate into active sites, with minimal diffusion limitations. As a result, the actual capacity approaches the theoretical nominal capacity, yielding a higher  value. Conversely, if , the discharge time is much shorter than the time constant of the rate-limiting process. This results in restricted transport processes within the electrolyte and a rate-limiting effect in the solid intercalation host, making lithium-ion intercalation more difficult and causing a decrease in capacity in the pristine electrode (Figure 4i). This observation aligns with the COMSOL simulation results, which show a more uniform lithium-ion concentration distribution on the electrode surface modified by the microneedle process.

CV data can be used to evaluate the electrochemical kinetics within a battery (Figure S3).^19,20^ At the same scan rate, higher peak current intensities (*I*_p_) indicate higher Li-ion diffusion coefficients (*D*_CV_), according to the Randles–Sevcik equation shown below^21^:
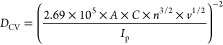
3

By plotting peak current (*I*_p_) against the square root of the scan rate (*v*^1/2^) (Figures S4), the Li-ion diffusion coefficient (*D*_CV_) in the electrode can be determined. Here, *A* is the electrode area (1.77 cm^2^), *C* is the molar concentration of lithium ions in the electrode (7.69 × 10^–3^ mol cm^–3^), and *n* is the number of electrons involved in the redox process (*n* = 1 for Fe^2+^/Fe^3+^). The diffusion rates of the punctured electrode during charging (oxidation) and discharging (reduction) are 9.30 × 10^–10^ and 3.98 × 10^–10^ cm^2^ s^–1^, respectively, approximately 1.3 and 1.5 times higher than those of the pristine electrode. This suggests that the low tortuosity structure provides a larger contact area between the electrode material and the electrolyte, facilitating deeper electrolyte penetration and easing Li-ion migration within the electrode.

**Figure 5:**
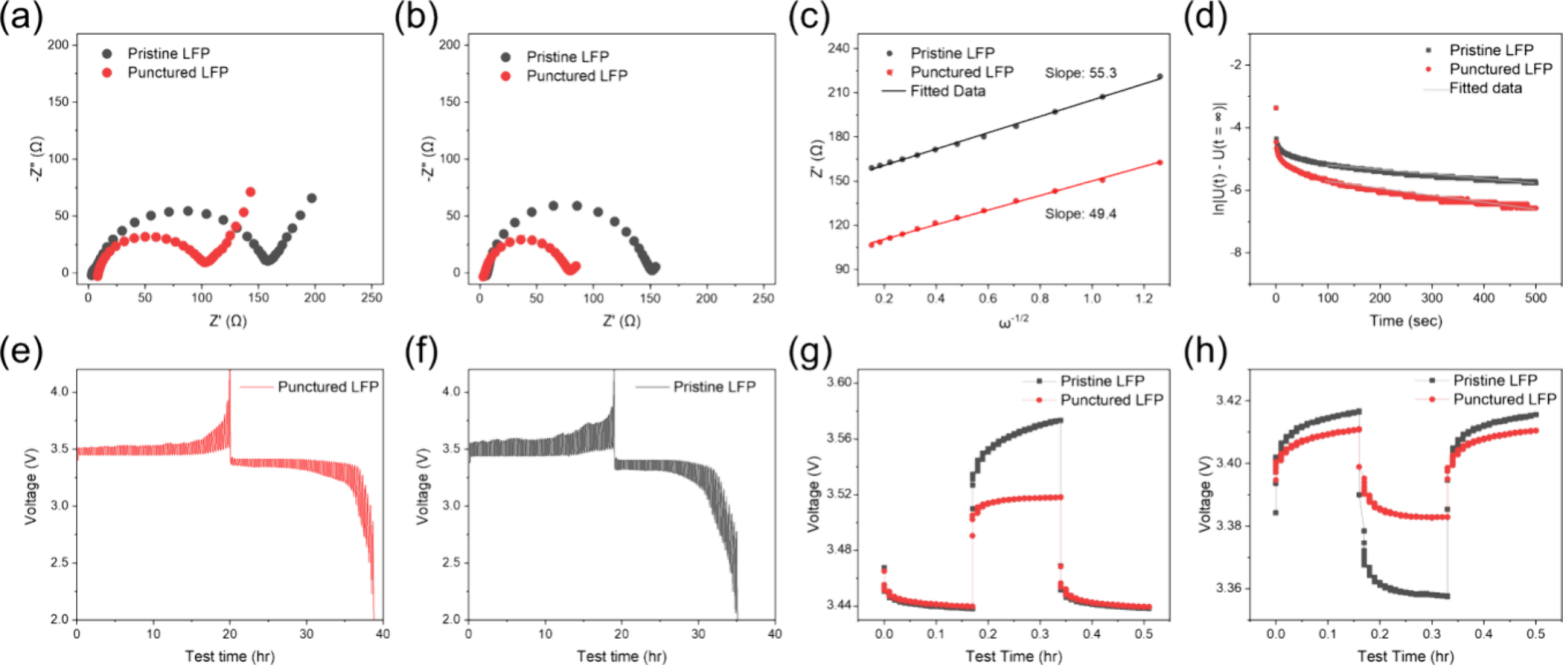
Nyquist plots of the punctured and pristine LFP electrodes (a) before cycling and (b) after cycling. (c) Linear relationship between Warburg impedance at low frequency and the inverse square root of angular frequency, with the slope representing the Warburg Factor. (d) ln|*U*(*t*) – U(*t* = ∞)| versus relaxation time plots showing the depolarization processes for the punctured and pristine LFP electrodes. GITT measurements of the (e) punctured LFP electrode and (f) pristine LFP electrode. Magnified view of single-step (g) charging and (h) discharging GITT curves.

To further verify the reasons for the improved electrochemical performance after the microneedle process, EIS analysis was conducted using an equivalent circuit model (Figure S5).^22^Figure 5a,b compares the impedance spectra of the LFP electrodes before and after cycling. It is evident that the punctured electrode exhibits lower resistance (*R*_e_ + *R*_CEI_ + *R*_ct_), which corresponds to the electrolyte, cathode electrode interphase (CEI),^23^ and charge transfer resistances, respectively. This improvement can be attributed to the low-curvature holes, which do not hinder the formation of a low-resistance CEI, while simultaneously enhancing Li-ion conduction and reducing charge transfer resistance. The detailed impedance values obtained from fitting are listed in Table S5, demonstrating that the microneedle process effectively enhances electrochemical performance. By fitting the Nyquist plots of the punctured and pristine LFP electrodes (Figure 5a), a sloped line can be observed at low frequencies, from which we can derive the Warburg factor.^24,25^ Using the following equation, we can calculate the diffusion coefficient (*D*_EIS_)^24^:
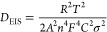
4

In this equation, *R* represents the gas constant (8.314 J mol^–1^ K^–1^), *T* is the temperature (298.5 K), *F* is the Faraday constant (96485 C mol^–1^), and σ can be obtained from the following equation^24^:
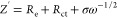
5

The slope of the simulated line, as shown in Figure 5c, is the Warburg constant (σ). Thus, the diffusion coefficient (*D*_EIS_) for the punctured electrode is approximately 7.86 × 10^–11^ cm^2^ s^–1^, which is about 30% higher than that of the pristine electrode. This indicates that the low-curvature holes effectively accelerate Li-ion migration.

Additionally, the DC-depolarization method can quantify the electrode resistance to Li-ion flux.^26,27^ First, an independent LFP electrode without aluminum foil is prepared using an etching process.^28^ This electrode is sandwiched between two separators and two layers of lithium foil. After injecting the electrolyte and resting for 10 h, a small current of 10 μA is applied for 2 h (polarization). This is followed by a relaxation process (depolarization) until the open circuit voltage (OCV) is reached, defined as the equilibrium potential (d*U*/d*t* <0.1 mV h^–1^). The equation used for fitting the linear region is^27^
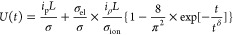
6

From this equation, the slope of the linear region during the depolarization process is used to obtain the characteristic relaxation time (*t*^δ^),^27^ as shown in Figure 5d. According to the experimental results, the slopes for the punctured and pristine electrodes are 1.26 × 10^–3^ and 8.01 × 10^–4^, respectively. The inverse of these slopes gives the relaxation times: *t*^δ^_punctured_ = 794 s and *t*^δ^_pristine_ = 1248 s. Using the formula:
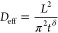
7where *L* is the electrode thickness (1.06 × 10^–2^ cm), the effective Li-ion diffusion coefficient (*D*_eff_) can be calculated for the electrodes. For the punctured electrode, *D*_eff_ is 1.43 × 10^–8^ cm^2^ s^–1^, which is 1.56 times higher than that of the pristine electrode.

The GITT technique was also used to study the Li-ion diffusion process in these electrodes.^19^ GITT testing involves a cycle of pulse-constant current-relaxation processes. The pulse refers to a short current application, while the relaxation refers to a resting period with no current. During a fixed period, a constant current is applied for charging or discharging, followed by an interruption of the current, and the voltage changes during both the constant current and relaxation periods are recorded. The pulse current is set to 0.1C, with the constant current and relaxation times controlled at 10 min each. The Li-ion diffusion coefficient (*D*_GITT_) is calculated using the following equation^8^:
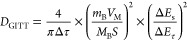
8

In this equation, *M*_B_ is 157.76 g mol^–1^, *V*_M_ is 46 cm^3^ mol^–1^, and  (0.0066 g cm^–2^) is the areal electrode loading. Comparisons of the GITT results for the punctured and pristine electrodes are shown in Figure 5e–h. The OCV at the end of the relaxation period, considered as the equilibrium potential for Li-ion intercalation, is around 3.42–3.44 V for these electrodes, confirming the characteristic of the LFP electrodes. The punctured electrode exhibits a smaller voltage plateau difference compared to the pristine electrode, as illustrated in Figure 5g,h, indicating less polarization. Based on the GITT curves, the charge/discharge Li-ion diffusion coefficients for these electrodes were calculated (Table S6). The punctured electrode shows significantly larger diffusion coefficients during both charging and discharging compared to the pristine electrode. This suggests that the low-tortuosity structure enhances Li-ion diffusion by providing additional channels between the electrode material and the electrolyte interfaces. These channels generated from the microneedle process facilitate efficient electrolyte penetration, thereby reducing the Li-ion migration barrier.

Previous studies have explored the differences in diffusion rates of LFP electrodes using three different analytical methods.^19,29^ However, as electrode thickness increases, EIS may not reach the linear diffusion region at lower frequencies, leading to some discrepancies in the diffusion rates calculated directly using the Warburg factor. Additionally, EIS-derived diffusion coefficients, being based on dynamic conditions, may not fully capture the slow kinetics of phase transitions. CV, on the other hand, has limitations in tracking changes in diffusion rates during the lithiation process, as it is based on assumptions of homogeneous diffusion, which may not accurately represent complex materials like LFP. GITT, by modeling in the relaxation region, can obtain lithium-ion diffusion rates that are closer to theoretical values. GITT is often considered more reliable than EIS for diffusion measurements in systems where near-equilibrium conditions can be achieved, as it allows for a direct assessment of chemical diffusion under controlled conditions.^29^ Therefore, in thicker electrodes, the diffusion rates obtained from GITT could be more accurate.

## 4 Conclusions

The study demonstrates that 3D low-tortuosity structures with low-curvature holes significantly enhance the electrochemical performance of LFP electrodes via the acupuncture-inspired microneedle process. Through various simulation and experimental techniques, including COMSOL simulations, SEM imaging, rate performance testing, and multiple diffusion coefficient measurements, the punctured electrodes exhibited superior Li-ion transport properties compared to pristine electrodes. The introduction of low-curvature holes via a microneedle process created additional channels for electrolyte penetration, enhancing ion transport kinetics and reducing concentration polarization. The punctured electrodes showed improved rate performance, maintaining higher specific capacities at various current rates, reaching 145 mAh g^–1^ at 0.1C and 101 mAh g^–1^ at 3C, outperforming the pristine electrode. Diffusion coefficient measurements further highlighted this improvement: CV analysis showed higher Li-ion diffusion coefficients for the punctured electrodes, approximately 1.3–1.5 times higher than the pristine electrodes, EIS analysis revealed a 30% higher diffusion coefficient for the punctured electrodes, DC-depolarization indicated an effective diffusion coefficient 1.56 times higher, and GITT testing confirmed significantly larger diffusion coefficients during both charging and discharging. Additionally, the study suggests that the microneedle process for creating low-tortuosity structures can be scaled up through automation, machine arm operation, and roll-to-roll manufacturing, making it feasible for large-scale production of high-performance electrodes. Overall, the incorporation of low-curvature holes in LFP electrodes significantly improves Li-ion diffusion and electrochemical performance, holding promise for the future development of high-power and high-energy-density batteries with potential applications in various fields requiring efficient energy storage solutions.

## Supporting Information Available

Additional experimental parameters, literature review summary, and test results (DOCX)

## References

[ref1] Kang C.-Y.; Su Y.-S. Smart Manufacturing Processes of Low-Tortuous Structures for High-Rate Electrochemical Energy Storage Devices. Micromachines 2022, 13 (9), 1534 10.3390/mi13091534. 36144156 PMC9500693

[ref2] Gu X.-X.; Kuang L.-Y.; Lin J.; Qiao S.; Ma S.; Li Y.; Wang Q.; Dai J.-H.; Zhou X.; Zhou H.-Y.; Chen T.-Z. Highly Porous Nitrogen-Doped Biochar Nanosheets for High-Performance Li–Se Batteries. Rare Met. 2023, 42 (3), 822–829. 10.1007/s12598-022-02163-2.

[ref3] Liao J.; Zhang X.; Zhang Q.; Hu Q.; Li Y.; Du Y.; Xu J.; Gu L.; Zhou X. Synthesis of KVPO_4_F/Carbon Porous Single Crystalline Nanoplates for High-Rate Potassium-Ion Batteries. Nano Lett. 2022, 22 (12), 4933–4940. 10.1021/acs.nanolett.2c01604. 35671041

[ref4] Xu Y.; Du Y.; Chen H.; Chen J.; Ding T.; Sun D.; Kim D. H.; Lin Z.; Zhou X. Recent Advances in Rational Design for High-Performance Potassium-Ion Batteries. Chem. Soc. Rev. 2024, 53 (13), 7202–7298. 10.1039/D3CS00601H. 38855863

[ref5] Wang J.; Xu Z.; Eloi J.; Titirici M.; Eichhorn S. J. Ice-Templated, Sustainable Carbon Aerogels with Hierarchically Tailored Channels for Sodium- and Potassium-Ion Batteries. Adv. Funct Materials 2022, 32 (16), 2110862 10.1002/adfm.202110862.

[ref6] Sander J. S.; Erb R. M.; Li L.; Gurijala A.; Chiang Y.-M. High-Performance Battery Electrodes via Magnetic Templating. Nat. Energy 2016, 1 (8), 16099 10.1038/nenergy.2016.99.

[ref7] Wang J.; Sun Q.; Gao X.; Wang C.; Li W.; Holness F. B.; Zheng M.; Li R.; Price A. D.; Sun X.; Sham T.-K.; Sun X. Toward High Areal Energy and Power Density Electrode for Li-Ion Batteries via Optimized 3D Printing Approach. ACS Appl. Mater. Interfaces 2018, 10 (46), 39794–39801. 10.1021/acsami.8b14797. 30372018

[ref8] Wu S.; Zheng H.; Wang X.; Zhang N.; Cheng W.; Fu B.; Chen H.; Liu H.; Duan H. High-Capacity, Low-Tortuosity LiFePO_4_-Based Composite Cathode Enabled by Self-Supporting Structure Combined with Laser Drilling Technology. Chemical Engineering Journal 2022, 430, 132810 10.1016/j.cej.2021.132810.

[ref9] Cho S.; Jang H. Y.; Jung I.; Liu L.; Park S. Synthesis of Embossing Si Nanomesh and Its Application as an Anode for Lithium Ion Batteries. J. Power Sources 2017, 362, 270–277. 10.1016/j.jpowsour.2017.07.048.

[ref10] Shen F.; Luo W.; Dai J.; Yao Y.; Zhu M.; Hitz E.; Tang Y.; Chen Y.; Sprenkle V. L.; Li X.; Hu L. Ultra-Thick, Low-Tortuosity, and Mesoporous Wood Carbon Anode for High-Performance Sodium-Ion Batteries. Adv. Energy Mater. 2016, 6 (14), 1600377 10.1002/aenm.201600377.

[ref11] Du Y.; Zhang Z.; Xu Y.; Bao J.; Zhou X. Metal Sulfide-Based Potassium-Ion Battery Anodes: Storage Mechanisms and Synthesis Strategies. Acta Phys. Chim. Sin. 2022, 38 (11), 2205017 10.3866/PKU.WHXB202205017.

[ref12] Nie L.; Chen S.; Zhang C.; Dong L.; He Y.; Gao T.; Yu J.; Liu W. Integration of a Low-Tortuous Electrode and an in-Situ-Polymerized Electrolyte for All-Solid-State Lithium-Metal Batteries. Cell Reports Physical Science 2022, 3 (4), 100851 10.1016/j.xcrp.2022.100851.

[ref13] Wang H.; Li J.; Miao Z.; Huang K.; Liao Y.; Xu X.; Meng J.; Li Z.; Huang Y. Enabling Ultrahigh-Capacity LiFePO_4_ Cathodes with Low Tortuosity. ACS Appl. Mater. Interfaces 2023, 15 (22), 26824–26833. 10.1021/acsami.3c04072. 37218051

[ref14] Zhang Y.; Shahriar M.; Hu S. Structuring Electrodes *via* Acoustic-Field-Assisted Particle Patterning for Enhanced Performance of Lithium-Ion Batteries. J. Mater. Chem. A 2023, 11 (22), 11849–11858. 10.1039/D3TA01180A.

[ref15] Moškon J.; Pivko M.; Gaberščk M. Basic Electrochemical Performance of Pure LiMnPO_4_: A Comparison with Selected Conventional Insertion Materials. ACSi 2016, 459–469. 10.17344/acsi.2015.2195. 27640373

[ref16] Dreyer W.; Jamnik J.; Guhlke C.; Huth R.; Moškon J.; Gaberšček M. The Thermodynamic Origin of Hysteresis in Insertion Batteries. Nat. Mater. 2010, 9 (5), 448–453. 10.1038/nmat2730. 20383130

[ref17] Shi B.; Shang Y.; Pei Y.; Pei S.; Wang L.; Heider D.; Zhao Y. Y.; Zheng C.; Yang B.; Yarlagadda S.; Chou T.-W.; Fu K. K. Low Tortuous, Highly Conductive, and High-Areal-Capacity Battery Electrodes Enabled by Through-Thickness Aligned Carbon Fiber Framework. Nano Lett. 2020, 20 (7), 5504–5512. 10.1021/acs.nanolett.0c02053. 32551672

[ref18] Heubner C.; Seeba J.; Liebmann T.; Nickol A.; Börner S.; Fritsch M.; Nikolowski K.; Wolter M.; Schneider M.; Michaelis A. Semi-Empirical Master Curve Concept Describing the Rate Capability of Lithium Insertion Electrodes. J. Power Sources 2018, 380, 83–91. 10.1016/j.jpowsour.2018.01.077.

[ref19] Tang K.; Yu X.; Sun J.; Li H.; Huang X. Kinetic Analysis on LiFePO_4_ Thin Films by CV, GITT, and EIS. Electrochim. Acta 2011, 56 (13), 4869–4875. 10.1016/j.electacta.2011.02.119.

[ref20] Ma S.; Wan G.; Yan Z.; Liu X.; Chen T.; Wang X.; Dai J.; Lin J.; Liu T.; Gu X. Eco-Friendly Aqueous Binder Derived from Waste Ramie for High-Performance Li-S Battery. Chin. Chem. Lett. 2024, 109853 10.1016/j.cclet.2024.109853.

[ref21] Huang Y.-H.; Wang F.-M.; Huang T.-T.; Chen J.-M.; Hwang B.-J.; Rick J. Micro-Electrode Linked Cyclic Voltammetry Study Reveals Ultra-Fast Discharge and High Ionic Transfer Behavior of LiFePO_4_. Int. J. Electrochem. Sci. 2012, 7 (2), 1205–1213. 10.1016/S1452-3981(23)13408-0.

[ref22] Dessantis D.; Di Prima P.; Versaci D.; Amici J.; Francia C.; Bodoardo S.; Santarelli M. Aging of a Lithium-Metal/LFP Cell: Predictive Model and Experimental Validation. Batteries 2023, 9 (3), 146 10.3390/batteries9030146.

[ref23] Zhang Z.; Yang J.; Huang W.; Wang H.; Zhou W.; Li Y.; Li Y.; Xu J.; Huang W.; Chiu W.; Cui Y. Cathode-Electrolyte Interphase in Lithium Batteries Revealed by Cryogenic Electron Microscopy. Matter 2021, 4 (1), 302–312. 10.1016/j.matt.2020.10.021.

[ref24] Xiao P.; Lv T.; Chen X.; Chang C. LiNi_0.8_Co_0.15_Al_0.05_O_2_: Enhanced Electrochemical Performance From Reduced Cationic Disordering in Li Slab. Sci. Rep 2017, 7 (1), 1408 10.1038/s41598-017-01657-9. 28469166 PMC5431203

[ref25] Ma S.; Liu X.; Chen T.; Wang Y.; Wang M.; Jiang F.; Zhou X.; Gu X. A Sustainable and Cost-Effective Nitrogen-Doped Three-Dimensional Porous Carbon for High-Performance Lithium-Sulfur Batteries. ChemSusChem 2024, e202400576 10.1002/cssc.202400576. 38823005

[ref26] Wu J.; Ju Z.; Zhang X.; Quilty C.; Takeuchi K. J.; Bock D. C.; Marschilok A. C.; Takeuchi E. S.; Yu G. Ultrahigh-Capacity and Scalable Architected Battery Electrodes *via* Tortuosity Modulation. ACS Nano 2021, 15 (12), 19109–19118. 10.1021/acsnano.1c06491. 34410706

[ref27] Li L.; Erb R. M.; Wang J.; Wang J.; Chiang Y. Fabrication of Low-Tortuosity Ultrahigh-Area-Capacity Battery Electrodes through Magnetic Alignment of Emulsion-Based Slurries. Adv. Energy Mater. 2019, 9 (2), 1802472 10.1002/aenm.201802472.

[ref28] Chen C.-H.; Chiu J.-M.; Shown I.; Wang C.-H. Simple Way of Making Free-Standing Cathode Electrodes for Flexible Lithium-Ion Batteries. RSC Adv. 2022, 12 (15), 9249–9255. 10.1039/D1RA08993E. 35424855 PMC8985147

[ref29] Wang J.; Koenig G. M. Comparison of Lithium Diffusion Coefficient Measurements in Tellurium Electrodes via Different Electrochemical Techniques. J. Electrochem. Soc. 2023, 170 (5), 050534 10.1149/1945-7111/acd43e.

