# Supplementary material for: High-Power Battery Electrodes Fabricated by Acupuncture-Inspired Microneedle Processing
Source: ACS Appl Mater Interfaces. 2024 Oct 3;16(41):55429–36. doi: 10.1021/acsami.4c11834 (PMC11492169; doi:10.1021/acsami.4c11834)
Supplement: Supplementary file 1 — am4c11834_si_001.docx [file am4c11834_si_001.docx]

**High-Power Battery Electrodes Fabricated by Acupuncture-Inspired Microneedle Processing**

Chun-Yang Kang^1^, Le-Yen Lin^2^, Thao Nguyen^3^, Chia-Chin Chen^2^, Jeng-Kuei Chang^4^, Tzu-En Lin^5^, and Yu-Sheng Su*^1,3^

^1^Industry Academia Innovation School, National Yang Ming Chiao Tung University, Hsinchu 300093, Taiwan

^2^Department of Chemical Engineering, National Taiwan University, Taipei 106319, Taiwan

^3^International College of Semiconductor Technology, National Yang Ming Chiao Tung University, Hsinchu 300093, Taiwan

^4^Department of Materials Science and Engineering, National Yang Ming Chiao Tung University, Hsinchu 300093, Taiwan

^5^Institute of Applied Mechanics, National Taiwan University, Taipei 106319, Taiwan

*Corresponding author: Yu-Sheng Su ([yushengsu@nycu.edu.tw](mailto:yushengsu@nycu.edu.tw))

|  | | |
| --- | --- | --- |
| **Parameters** | **Description** | **Value** |
| D_ion_ (m^2^ s^−1^) | Ionic diffusion coefficient in the electrolyte | 2×10^−10^ |
| t_Li_^+^ | Lithium-ion transference number in the electrolyte | 0.26 |
| c_Electrolyte_ (mol m^−3^) | Electrolyte salt concentration | 1000 |
| Electrolyte | Volume fraction of electrolyte in porous cathode | 0.4 |
| LFP | Volume fraction of LiFePO_4_ in porous cathode | 0.6 |
| e (S m^−1^) | Effective electronic conductivity of the cathode | 10 |
| D_Li_ (m^2^ s^−1^) | Lithium diffusion coefficient in LiFePO_4_ | 3×10^−16^ |
| r_LFP_ (m) | Average diameter of LiFePO_4_ particle | 1.2×10^−6^ |
| i_0, cathode_ (A m^−2^) | Exchange current density for cathode | 100 |
| i_0, anode_ (A m^−2^) | Exchange current density for anode | 100 |
| c | Cathodic transfer coefficient | 0.5 |
| a | Anodic transfer coefficient | 0.5 |

**Table S1.** COMSOL simulation parameter settings.

| **Areal Loading (mg cm^−2^)**  **@LFP wt.%** | **Capacity (mAh g^−1^) @Rate** | **Cycle Retention (%) @Cycle #** | **Technique** | **Ref.** |
| --- | --- | --- | --- | --- |
| 65@77% | 61@1C | 77%@80 | Phase Inversion | 1 |
| 20@37% | 108@10C | 88%@250 | Phase Inversion | 2 |
| 128@73% | 123@5 mA cm^-2^ | 68%@150 | Aligned Framework | 3 |
| 30@80% | 78@3C | 100%@120 | Magnetic Field | 4 |
| 11@87% | 54@3C | 78%@50 | Stamping | 5 |
| 18@85% | 55@5C | 72%@200 | Acoustic Field | 6 |
| 18@74% | 75@3.5C | 72%@315 | Electrostatic Self-assembly | 7 |
| 60@72% | 100@1C | 95%@80 | Template with Puncher | 8 |
| 12@73% | 94@2C | 81%@100 | Laser Drilling | 9 |
| 15.7@90% | 81@2C | 100%@75 | Microneedle | This Work |

**Table S2.** Comparison of recently developed thick and porous LFP electrodes for LIBs.

| **Type** | $\boldsymbol{\eta}_{\boldsymbol{Total}}$ **(V)** | $\boldsymbol{\eta}_{\boldsymbol{+}}$**(V)** | $\boldsymbol{\eta}_{\boldsymbol{-}}$ **(V)** |
| --- | --- | --- | --- |
| Punctured LFP | 0.29 | 0.14 | −0.15 |
| Pristine LFP | 0.74 | 0.26 | −0.48 |

**Table S3.** The overpotentials obtained from the voltage-time curves.

| **Type** | $\boldsymbol{\beta}$ | $\boldsymbol{\tau}_{\boldsymbol{fit}}$ **(h)** |
| --- | --- | --- |
| Punctured LFP | 0.49 | 0.36 |
| Pristine LFP | 0.8 | 0.69 |

**Table S4.** The fitting parameters obtained from the semi-empirical master curves.

| **Type** | **Cycle** | **R_e_ (Ω)** | **R_CEI_ (Ω)** | **R_ct_ (Ω)** |
| --- | --- | --- | --- | --- |
| Punctured LFP | Before | 2.1 | - | 87.8 |
| Pristine LFP | Before | 3.2 | - | 148.7 |
| Punctured LFP | After | 3.8 | 2.2 | 72.4 |
| Pristine LFP | After | 6.4 | 13.1 | 130.4 |

**Table S5.** The resistances obtained from the EIS spectra.

| **Type** | **Condition** | $\boldsymbol{D}_{\boldsymbol{GITT}}$ **(cm^2^ s^−1^)** |
| --- | --- | --- |
| Punctured LFP | Charge | 7.05 × 10^-12^ |
| Pristine LFP | Charge | 2.74 × 10^-14^ |
| Punctured LFP | Discharge | 2.89 × 10^-12^ |
| Pristine LFP | Discharge | 1.62 × 10^-12^ |

**Table S6.** The diffusion coefficients obtained from the GITT analysis.


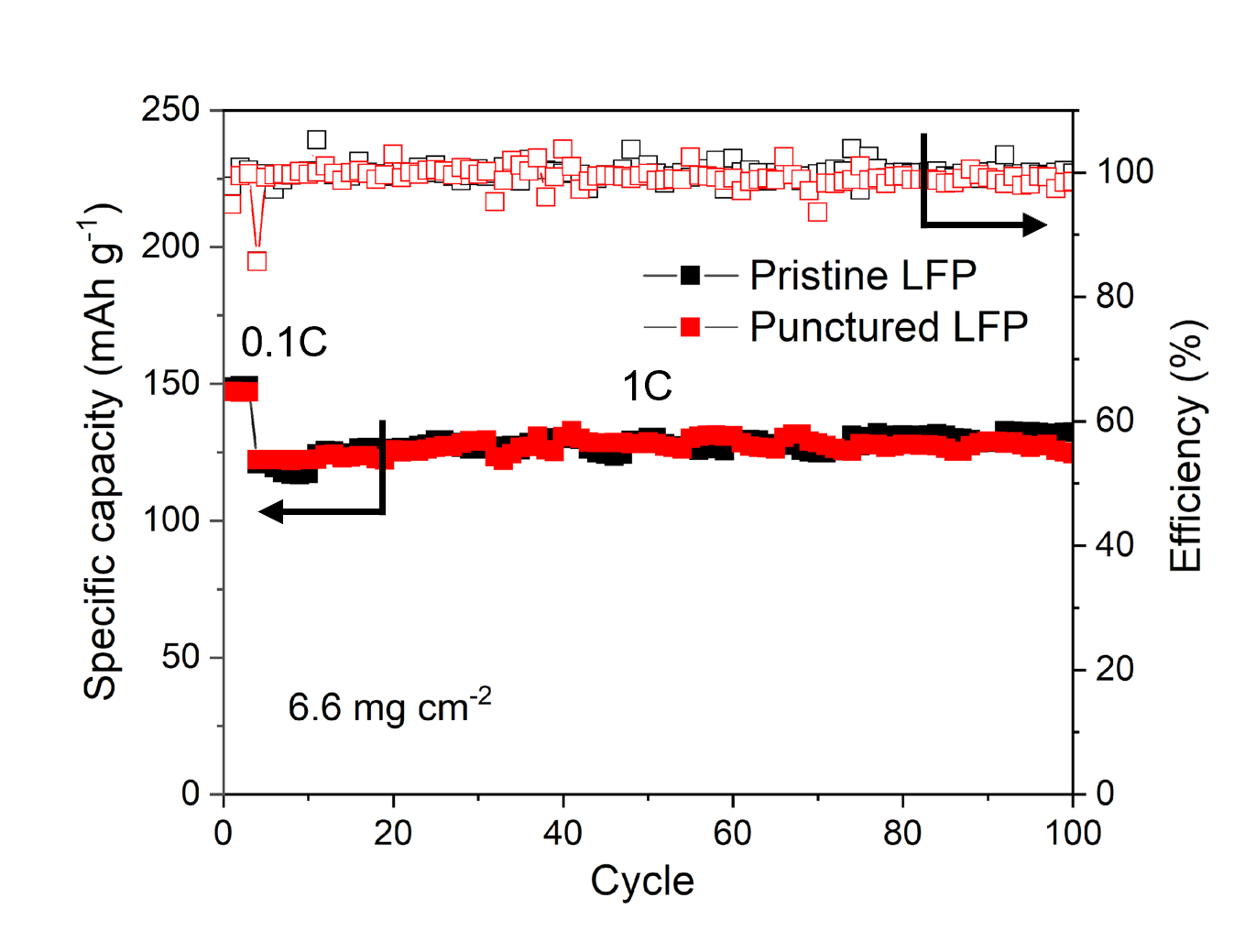


**Figure S1.** Cycle life data of the punctured LFP electrodes (first three cycles: 0.1C; then 1C).


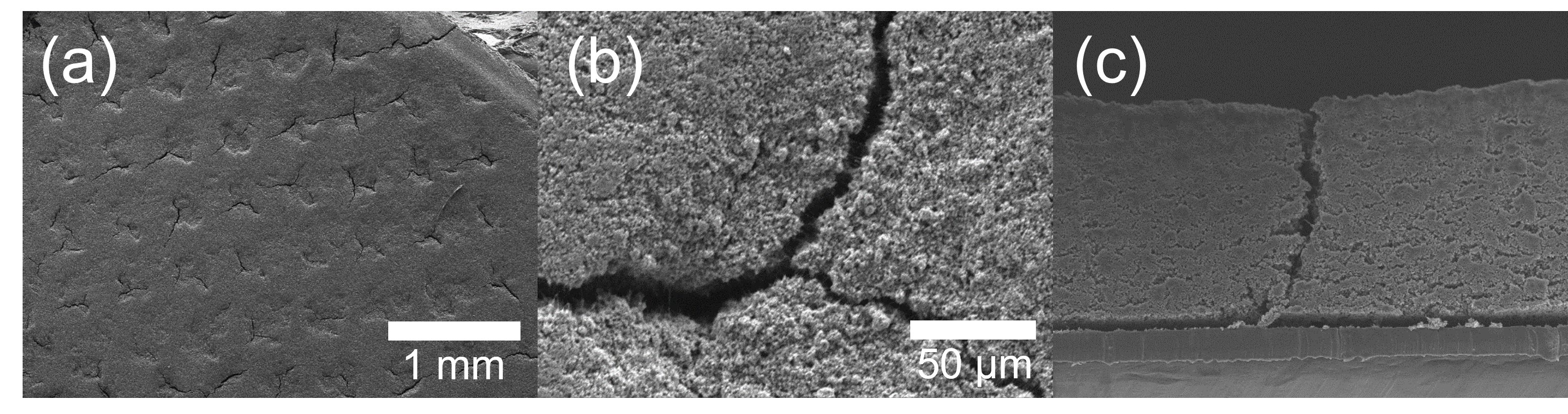


**Figure S2.** SEM images of the punctured LFP electrode from (a,b) top view and (c) side view after cycling.


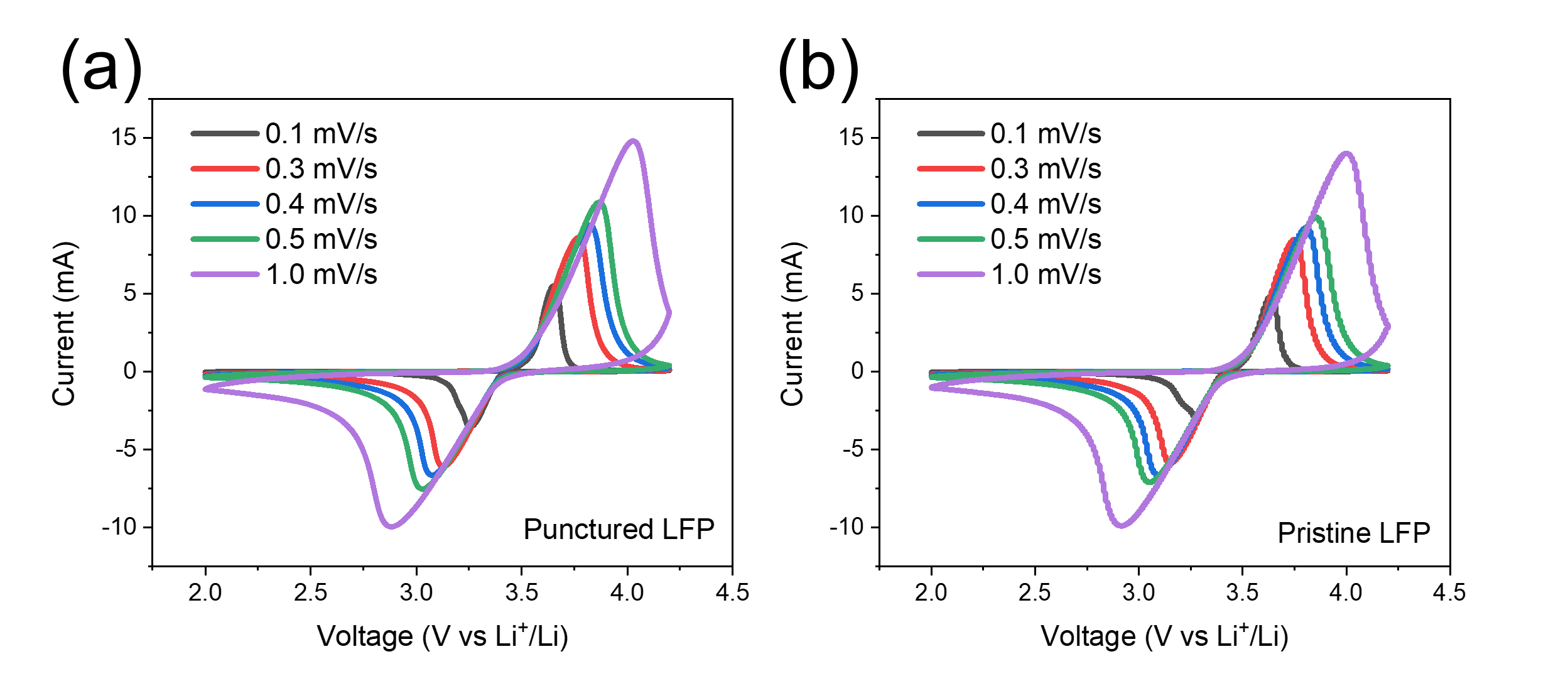
**Figure S3.** CV plots of the (a) punctured LFP electrode and (b) pristine LFP electrode at various sweep rates.


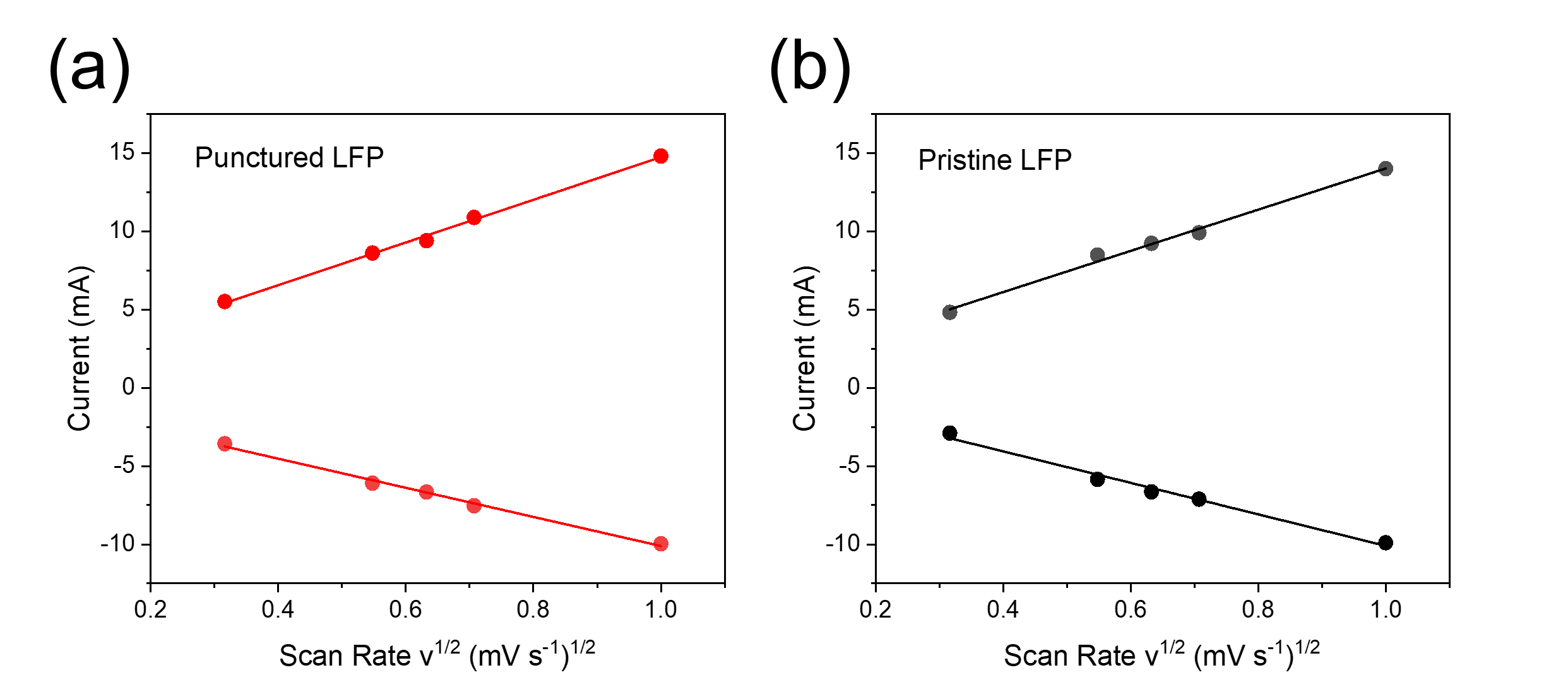


**Figure S4.** The relationship between $I_{p}$​ and $v^{1/2}$ obtained from the CV curves measured using the (a) punctured LFP electrode and (b) pristine LFP electrode.


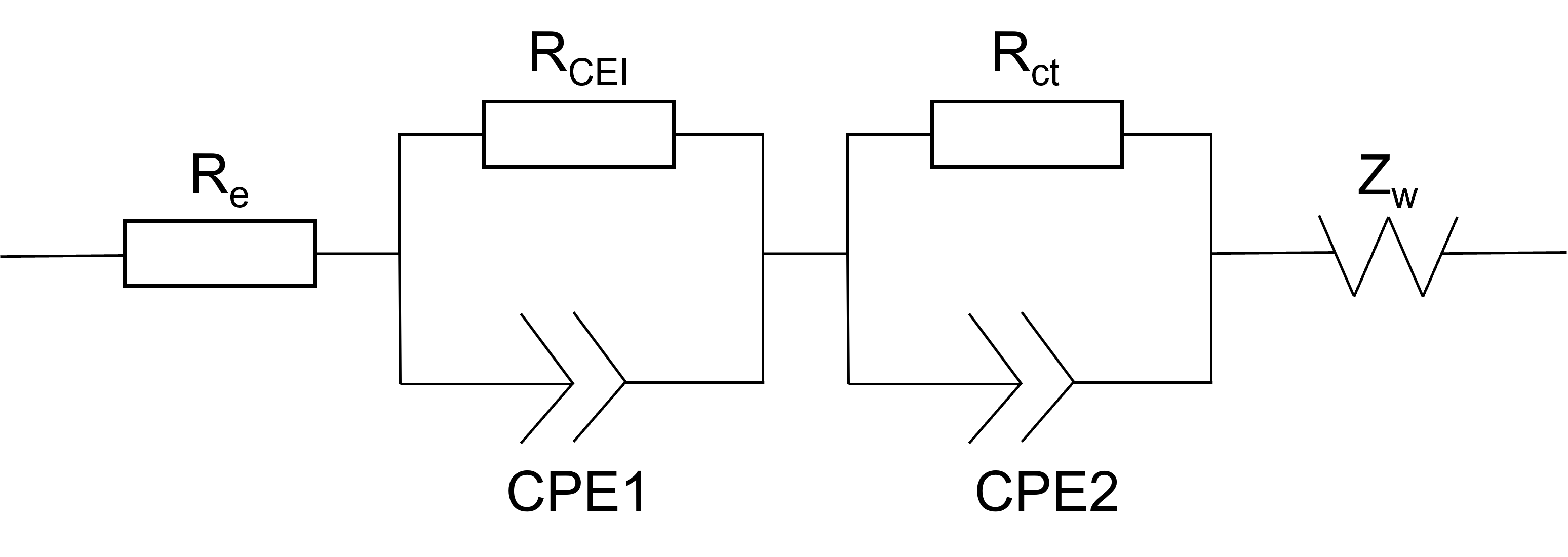


**Figure S5.** The EIS fitting equivalent circuit model.

**REFERENCES**

(1) Wu, J.; Ju, Z.; Zhang, X.; Takeuchi, K. J.; Marschilok, A. C.; Takeuchi, E. S.; Yu, G. Building Efficient Ion Pathway in Highly Densified Thick Electrodes with High Gravimetric and Volumetric Energy Densities. *Nano Lett.* **2021**, *21* (21), 9339–9346. https://doi.org/10.1021/acs.nanolett.1c03724.

(2) Wang, J.; Wang, M.; Ren, N.; Dong, J.; Li, Y.; Chen, C. High-Areal-Capacity Thick Cathode with Vertically-Aligned Micro-Channels for Advanced Lithium Ion Batteries. *Energy Storage Materials* **2021**, *39*, 287–293. https://doi.org/10.1016/j.ensm.2021.04.030.

(3) Shi, B.; Shang, Y.; Pei, Y.; Pei, S.; Wang, L.; Heider, D.; Zhao, Y. Y.; Zheng, C.; Yang, B.; Yarlagadda, S.; Chou, T.-W.; Fu, K. K. Low Tortuous, Highly Conductive, and High-Areal-Capacity Battery Electrodes Enabled by Through-Thickness Aligned Carbon Fiber Framework. *Nano Lett.* **2020**, *20* (7), 5504–5512. https://doi.org/10.1021/acs.nanolett.0c02053.

(4) Wu, J.; Ju, Z.; Zhang, X.; Xu, X.; Takeuchi, K. J.; Marschilok, A. C.; Takeuchi, E. S.; Yu, G. Low-Tortuosity Thick Electrodes with Active Materials Gradient Design for Enhanced Energy Storage. *ACS Nano* **2022**, *16* (3), 4805–4812. https://doi.org/10.1021/acsnano.2c00129.

(5) Ren, H.; Wang, Y.; Cao, D.; Gedney, W.; Ji, T.; Sun, X.; Zhu, H. Manufacturing Water‐Based Low‐Tortuosity Electrodes for Fast‐Charge through Pattern Integrated Stamping. *Energy &amp; Environ Materials* **2023**, *6* (4), e12584. https://doi.org/10.1002/eem2.12584.

(6) Zhang, Y.; Shahriar, M.; Hu, S. Structuring Electrodes *via* Acoustic-Field-Assisted Particle Patterning for Enhanced Performance of Lithium-Ion Batteries. *J. Mater. Chem. A* **2023**, *11* (22), 11849–11858. https://doi.org/10.1039/D3TA01180A.

(7) Li, H.; Peng, L.; Wu, D.; Wu, J.; Zhu, Y.-J.; Hu, X. Ultrahigh-Capacity and Fire-Resistant LiFePO_4_-Based Composite Cathodes for Advanced Lithium-Ion Batteries. *Adv. Energy Mater.* **2019**, *9* (10), 1802930. https://doi.org/10.1002/aenm.201802930.

(8) Wang, H.; Li, J.; Miao, Z.; Huang, K.; Liao, Y.; Xu, X.; Meng, J.; Li, Z.; Huang, Y. Hierarchical Electrode Architecture Enabling Ultrahigh-Capacity LiFePO_4_ Cathodes with Low Tortuosity. *ACS Appl. Mater. Interfaces* **2023**, *15* (22), 26824–26833. https://doi.org/10.1021/acsami.3c04072.

(9) Wu, S.; Zheng, H.; Wang, X.; Zhang, N.; Cheng, W.; Fu, B.; Chen, H.; Liu, H.; Duan, H. High-Capacity, Low-Tortuosity LiFePO_4_-Based Composite Cathode Enabled by Self-Supporting Structure Combined with Laser Drilling Technology. *Chemical Engineering Journal* **2022**, *430*, 132810. https://doi.org/10.1016/j.cej.2021.132810.
